# Dose-Response Mixed Models for Repeated Measures – a New Method for Assessment of Dose-Response

**DOI:** 10.1007/s11095-020-02882-0

**Published:** 2020-07-31

**Authors:** Gustaf J. Wellhagen, Bengt Hamrén, Maria C. Kjellsson, Magnus Åstrand

**Affiliations:** 1 Clinical and Quantitative Pharmacology, Clinical Pharmacology and Safety Sciences, R&D, AstraZeneca, Gothenburg, Sweden; 2 Department of Pharmaceutical Biosciences, Uppsala University, Uppsala, Sweden

**Keywords:** Mixed models for repeated measures (MMRM), dose-response analysis, dose-response mixed models for repeated measures (DR-MMRM), chronic kidney disease (CKD), urinary albumin-to-creatinine ratio (UACR)

## Abstract

**Purpose:**

In this paper we investigated a new method for dose-response analysis of longitudinal data in terms of precision and accuracy using simulations.

**Methods:**

The new method, called Dose-Response Mixed Models for Repeated Measures (DR-MMRM), combines conventional Mixed Models for Repeated Measures (MMRM) and dose-response modeling. Conventional MMRM can be applied for highly variable repeated measure data and is a way to estimate the drug effect at each visit and dose, however without any assumptions regarding the dose-response shape. Dose-response modeling, on the other hand, utilizes information across dose arms and describes the drug effect as a function of dose. Drug development in chronic kidney disease (CKD) is complicated by many factors, primarily by the slow progression of the disease and lack of predictive biomarkers. Recently, new approaches and biomarkers are being explored to improve efficiency in CKD drug development. Proteinuria, i.e. urinary albumin-to-creatinine ratio (UACR) is increasingly used in dose finding trials in patients with CKD. We use proteinuria to illustrate the benefits of DR-MMRM.

**Results:**

The DR-MMRM had higher precision than conventional MMRM and less bias than a dose-response model on UACR change from baseline to end-of-study (DR-EOS).

**Conclusions:**

DR-MMRM is a promising method for dose-response analysis.

**Electronic supplementary material:**

The online version of this article (10.1007/s11095-020-02882-0) contains supplementary material, which is available to authorized users.

## Background

Traditional dose-response analyses are strongly dependent on the choice of model when the response is highly variable due to unexplained variability. Model-based analyses give higher statistical power than group-wise comparisons [1]. However, many alternative models might provide similar predictions. To select the most appropriate model can be challenging when the signal-to-noise ratio (SNR) is low, since the knowledge that can be gained is proportional to the SNR [2, 3]. The model uncertainty results in large sample size and/or uncertainty in the dose selection for the following study.

Mixed Models for Repeated Measures (MMRM) is an approach to model data with high unexplained variability, making few/no assumptions regarding the response. Instead, at each visit a placebo and treatment response is estimated independent of other visits. This approach has proved superior in terms of precision and accuracy to analysis of (co)variance (ANOVA/ANCOVA) with the end-of-study data in cases with dropout, where the traditional alternative is to use last observation carried forward (LOCF) [4–11].

As an ANCOVA is only based on the information from the last visit, it does not make use of the totality of data. The conventional MMRM technique also ignores some of the information in the data, since each visit and dose level is modeled independently as a factor. By assuming a dose-response relationship where the dose is handled as a continuous variable, information can be shared between dose arms to improve prediction accuracy. Dose-response analysis is most commonly applied to end-of-study data, thus ignoring shared information across visits. In this manuscript we present a new method for dose-response analysis of longitudinal data. The new method, Dose-Response Mixed Models for Repeated Measures (DR-MMRM), combines conventional MMRM and dose-response modeling, with few assumptions regarding the response but sharing information across doses and visits. In DR-MMRM each visit has a separate placebo and E_max_ estimate, while ED_50_ is a global parameter. Similar methods have been described previously, but then focused on exposure-response analysis of QT interval prolongation [12, 13]. These studies also handled E_max_ as a global parameter, while DR-MMRM can account for different time-courses of the drug effect.

Chronic kidney disease (CKD) is a global burden, which was estimated to cause 1.2 million deaths in 2015, an increase of over 30% compared to 2005 [14]. The disease progression of CKD is often slow with few initial symptoms. Traditionally, drug development in CKD has focused on estimated glomerular filtration rate (eGFR). It is recognized that clinical trials within the CKD field face many challenges, related to the slow progression of the disease but also the selection of sensitive clinical endpoint, patient recruitment and influence of comorbidities [15]. Recently, the National Kidney Foundation (NKF), held a workshop together with the FDA and EMA where new approaches to improve efficiency in CKD drug development were discussed [16]. New opportunities were highlighted including the validity of urinary albumin-to-creatinine ratio (UACR) as an important clinical endpoint [16]. UACR is commonly used as a dose-finding clinical endpoint in CKD, instead of eGFR, because it typically has a faster response that allows for shorter studies [16, 17]. However, UACR is highly variable both between and within subjects. The new method, DR-MMRM, was applied to simulated UACR as a clinical endpoint to demonstrate its usefulness.

## Objectives

To investigate the precision and accuracy of placebo-adjusted change from baseline of simulated UACR (∆∆UACR) with different methods: dose-response on end-of-study data (DR-EOS), conventional MMRM and dose-response MMRM (DR-MMRM).

## Methods

### Previous Studies

Inspiration for the simulation study setup and the estimates of variability and correlation between residuals were taken from a previous study of dapagliflozin where UACR was measured over time (data on file, *n* = 251). This was a three-arm, double-blinded, placebo-controlled, parallel group, randomized clinical trial in a population with type 2 diabetes and mild renal impairment. Scheduled visits occurred at baseline and weeks 1, 2, 4, 6, 8, 10, 12, 14, 16, 18, 20, 22, and 24. Several models for the correlation of residuals (autoregressive of different order, independence, etc.) in the placebo arm of this study (*n* = 84) were evaluated and compared based on Akaike Information Criterion (AIC) and Bayesian Information Criterion (BIC) as well as by inspecting the model population predictions. As all models provided similar fit (not shown), a 1st order autoregressive model was chosen for simulations.

### Simulations

**Fig. 1:**
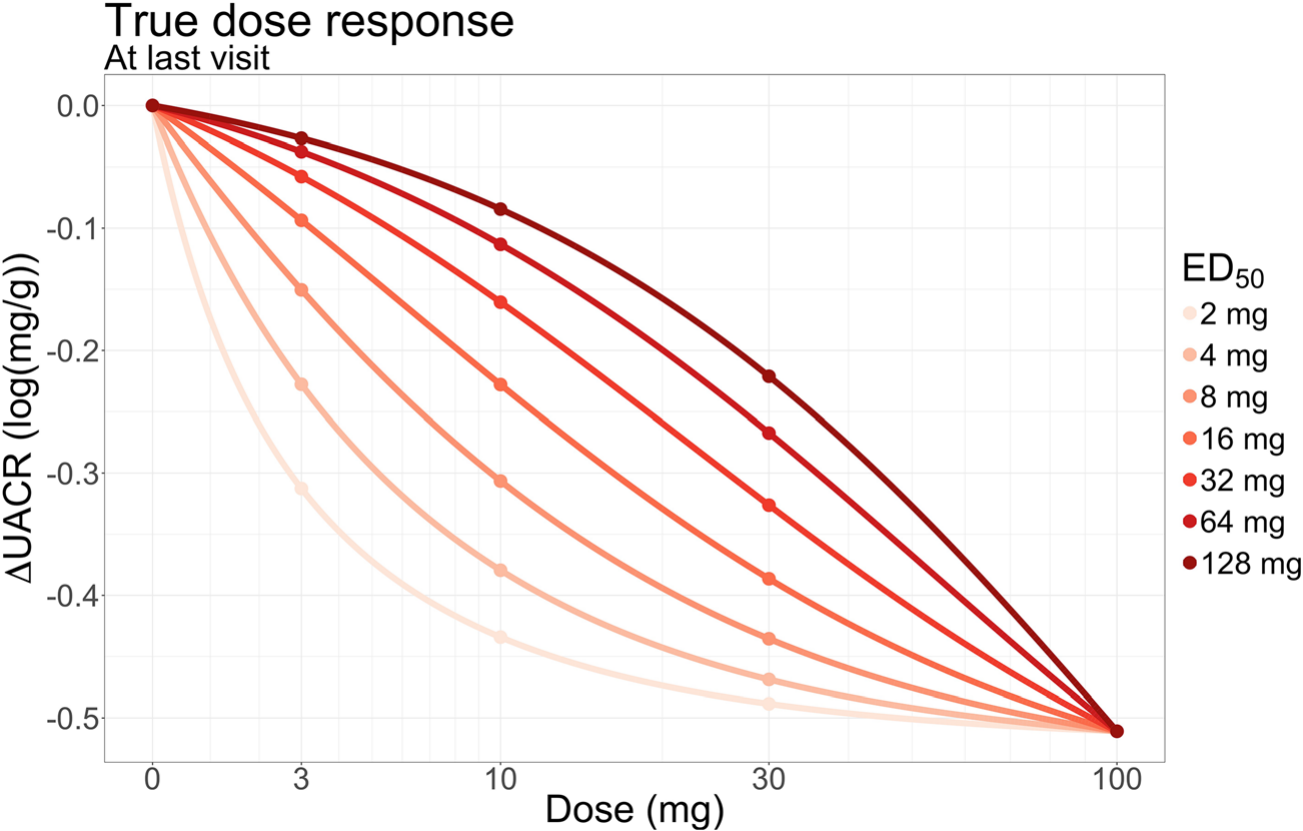
True dose-response relationship used in the simulations. The effect is shown at the last visit

The model for the previous data was used as a basis for the simulations. A true dose-response model following an E_max_ shape with a range of ED_50_ (2–128 mg) was assumed, see Fig. 1. The E_max_ parameter was set so that the highest dose achieved a reduction of 40% in UACR at the last visit. The baseline UACR was set to a mean of 5.63 log(mg/g) and the baseline variability (ω) was set to 0.3716 log(mg/g), as found in the previous study. As the simulated values were change from baseline, the actual value of the baseline had no importance for the results. A 1st order autoregressive model (AR [1]) with ρ = 0.226/visit and a residual error magnitude (σ) of 0.50 log(mg/g) was assumed for the correlation of residuals, again based on previous findings.

The simulated values of UACR were log-transformed changes from baseline (∆UACR). Three different time-courses of the drug effects were investigated: direct, exponential or linear. For each combination of ED_50_ and drug effect time-course, a number (*n* = 1000) of 16-week studies were simulated, totaling 21,000 studies. Samples were taken at week −2, −1, 0, 2, 4, 6, 8, 10, 12, 14, 15 and 16. There was 1 placebo arm and 3 or 4 dose arms (10, 30 and 100 mg or 3, 10, 30 and 100 mg). The baseline was averaged across the visits at week −2, −1 and 0.

The sample was size set to provide 95% statistical power for detecting a 40% reduction in ∆UACR between the highest dose arm and placebo at end-of-study using a *t*-test, given the variability that was previously assumed. This led to the same sample size (*n* = 39 per arm) in all trials, while varying the E_max_ parameter at the end-of-study visit.

The E_max_ parameter also changed with respect to different time-courses of the drug effect. For the exponential and linear time-courses the 40% reduction was reached at the last visit with the highest dose. The direct effect-profile reached the 40% reduction already at the first visit post baseline with the highest dose. The following equations (Eq. 1–2) were used to calculate the E_max_ at each visit for each ED_50_ and time-course:

1 $$ {E}_{\mathit{\max},k}=\mathit{\log}(0.6)\times \frac{100+{ED}_{50,k}}{100} $$

2 $$ {E}_{\mathit{\max},k,t}=\left\{\begin{array}{c}{E}_{\mathit{\max},k}\times 1,\kern0.5em Direct\ effect\\ {}{E}_{\mathit{\max},k}\times \left(1-{e}^{-\frac{\log (2)}{1.75}\times t}\right),\kern0.5em Exponential\ effect\\ {}{E}_{\mathit{\max},k}\times \frac{t}{16},\kern0.5em Linear\ effect\end{array}\right. $$

where *E*_*max,k,t*_ is the change in UACR with ED_50,k_ (2, 4, 8, 16, 32, 64 or 128 mg) at *t* weeks after start of treatment.

### Models

Three methods were investigated. First, the dose-response relationship on change from baseline at end-of-study data was fitted only on change from baseline data at the last visit (week 16). Then, the placebo response, ED_50_ and E_max_ were estimated. Secondly, in the conventional MMRM analysis, each visit and dose arm had a separate estimate of both the placebo response and the response in each of the different dose arms. No dose-response through ED_50_ or E_max_ was estimated then. Lastly, in the dose-response MMRM analysis, each visit had a separate estimate of the placebo response and E_max_ but all visits shared the ED_50_ parameter. This saved 2 or 3 parameters per visit (depending on number of dose arms) but added 1 global. The MMRM methods were estimated with a 1st order autoregressive covariance matrix for the correlation of residuals.

A schematic of the simulation and estimation setup is shown in Appendix Fig. 1.

### Evaluation Metrics

The precision was assessed through the magnitude of the uncertainty of the estimated ∆∆UACR. Precision was also evaluated in the same way for the E_max_ and ED_50_ estimates in the dose-response informed methods that contained these parameters (DR-EOS and DR-MMRM).

The accuracy of ∆∆UACR was assessed through relative bias, computed as the absolute bias at the last visit compared to the true values, relative to the maximal effect (40% reduction of UACR). The bias for ED_50_ and E_max_ was assessed through absolute bias for the dose-response informed methods.

For ∆∆UACR, the root mean squared error (RMSE), which is a weighted measure of precision and accuracy, was also calculated at the last visit through Eq. 3:

3 $$ RMSE=\sqrt{sd^2+{bias}^2} $$

### Software and Implementation

Both simulations and estimations were performed in R version 3.2.4 [18]. The autocorrelation models of previous studies were fit using the nlme() package with the lme() function [19]. The nlme() package was also used to fit the dose-response MMRM relationship and the MCPMod() package was used to fit the dose-response relationship on end-of-study data [20].

In order to directly get the standard deviation and partly to stabilize the model, the dose-response MMRM was parameterized so that the estimates from each dose arm were obtained separately and the standard E_max_ equation was changed so the effect was given at dose = 1 – here called E_dose_ – instead of E_max_, reformulating the model as in Eq. 4:

4 $$ \boldsymbol{y}=\boldsymbol{Plc}+{\boldsymbol{E}}_{\boldsymbol{dose}}\times \frac{dose}{ED_{50}+ dose}\times \frac{ED_{50}+{dose}_m}{dose_m} $$

where **y** are the responses, **Plc** the placebo responses and dose_m_ is the dose in question (3, 10, 30 or 100 mg), collapsing to Eq. 5 when dose = dose_m_:

5 $$ \boldsymbol{y}=\boldsymbol{Plc}+{\boldsymbol{E}}_{\boldsymbol{dose}} $$

The estimates were only saved when dose = dose_m_ was fulfilled. This meant fitting the same data 3 or 4 times to get estimates for each dose arm, while obtaining the same estimate for ED_50_ – and also E_max_, which could be back-calculated. The number of times the models could not converge was recorded. Since the dose-response MMRM was performed with several attempts, a complete convergence failure was defined as when all parameterizations for one study failed simultaneously.

For full details on the implementation, R code with a working example is provided in Appendix File 1.

## Results

**Fig. 2:**
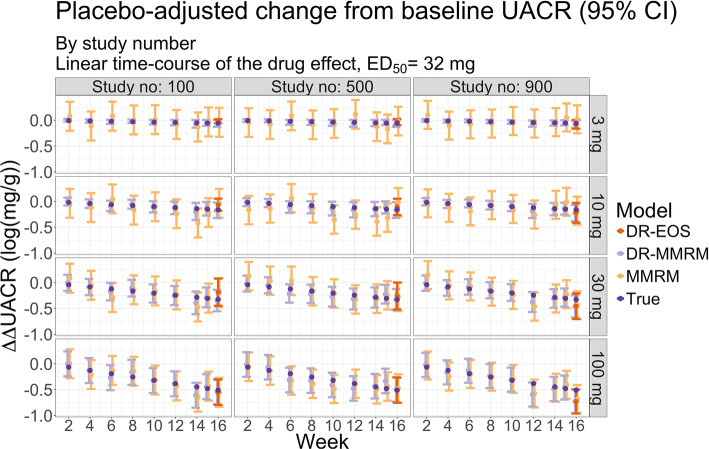
Placebo-adjusted ∆UACR and 95% CI for 3 studies with linear time-course of the drug effect where ED_50_ = 32 mg, stratified by dose. The true ∆∆UACR is also shown

The true and estimated ∆∆UACR with 95% confidence intervals for 3 example studies with linear drug effect time-course and ED_50_ = 32 mg are shown in Fig. 2. Due to the large number of simulated scenarios, the ∆∆UACR for more cases are not shown. The illustrational example in Fig. 2 shows how all 3 methods are comparable in precision for the highest dose at the end-of-study visit. The dose-response informed methods have shorter confidence intervals for the lower doses, and the DR-MMRM has the highest precision (lowest standard errors) in all cases. The same figure, but for the simulation scenario where only 3 doses and placebo were simulated is shown in Appendix Fig. 2.

**Fig. 3:**
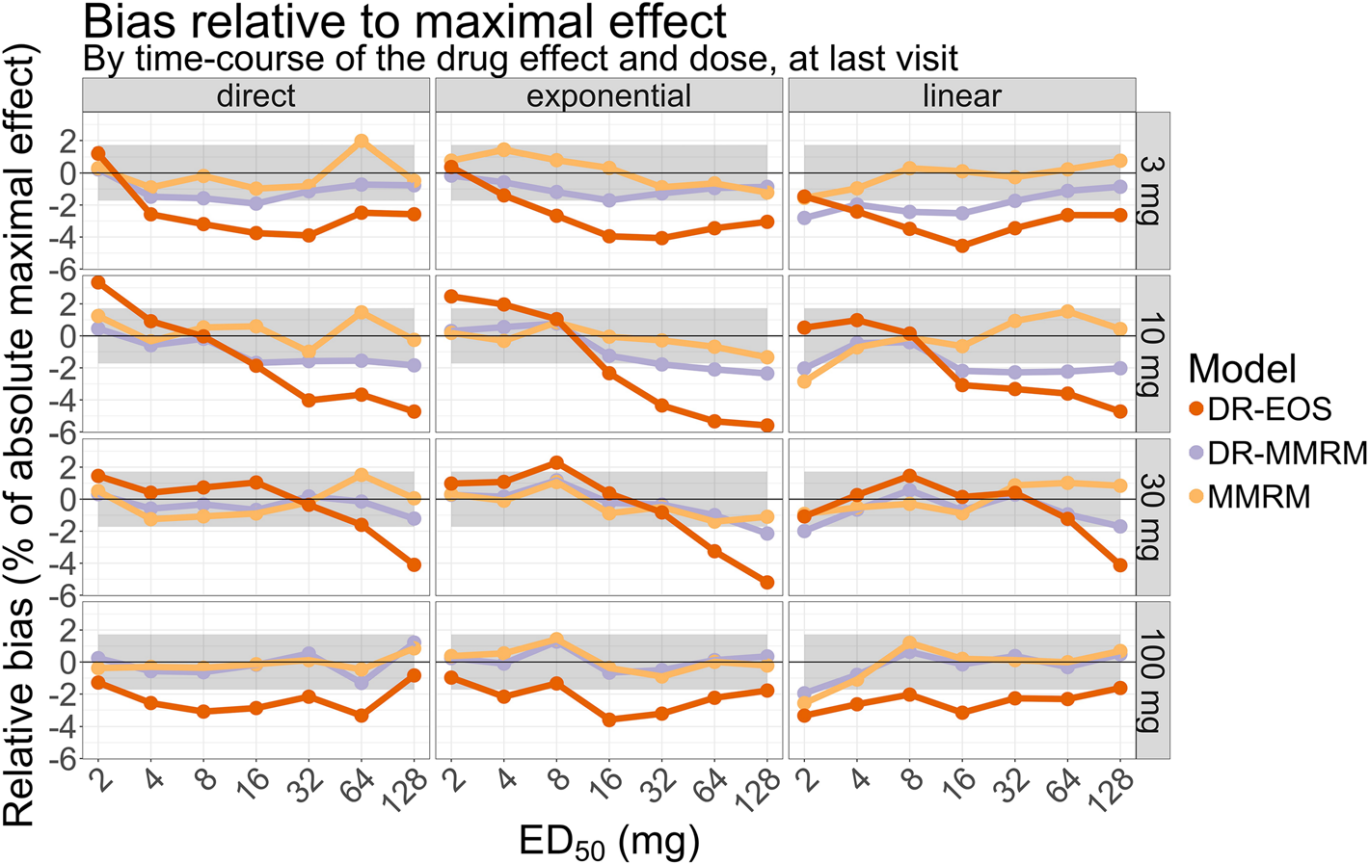
Bias relative to the absolute maximal effect at last visit, stratified by time-course of the drug effect and dose level. The gray area (±1.7%) indicates the expected variability (±2 SD) of estimates known to be unbiased given the simulation setup of 1000 simulations for each scenario

In Fig. 3, the bias at the last visit is shown in relation to the maximal effect (a reduction of 40% in UACR), stratified by time-course of the effect and the dose levels. The gray area indicates the expected variability (±2 SD) of estimates known to be unbiased given the simulation setup. The conventional MMRM is unbiased (if any dropout is at random) [4, 8] and generally showed estimated bias within the gray area which was the expected outcome: only 3 of 84 estimates (4%) fell outside this range. The expected outcome was 5%. All methods had low bias for the highest dose. For the dose-response informed methods, the bias increased with increasing ED_50_ which may be due to that the studied dose range (3–100 mg) covered a smaller part of the true dose-response relationship. The DR-MMRM had 18 of 84 estimates (21%) outside the expected theoretical range. The DR-EOS always had the strongest bias, which at most was around 6% of the maximal UACR effect, and 53 of 84 estimates (63%) had larger bias than the expected theoretical range. For DR-MMRM the highest bias was around 3% of the maximal UACR effect.

**Table 1:** Summary of pros and cons for the investigated methods

	Pros	Cons
DR-EOS	Simple, can be used for extrapolation or interpolation	Disregards information from all other visits
MMRM	Always unbiased, uses information from all visits	Parameter heavy, cannot extrapolate or interpolate
DR-MMRM	Makes use of shared information from all visits and dose arms, higher ED_50_ precision than DR-EOS, can be used for extrapolation or interpolation, overall best precision of ∆∆UACR	Assumes the same ED_50_ for all visits

DR-EOS, Dose-Response at End-of-Study; DR-MMRM, Dose-Response Mixed Models for Repeated Measures; ED_50_, effective dose giving 50% of maximal effect; MMRM, Mixed Models for Repeated Measures; ∆∆UACR, Placebo-adjusted change from baseline of Urinary Albumin-to-Creatinine Ratio

**Fig. 4:**
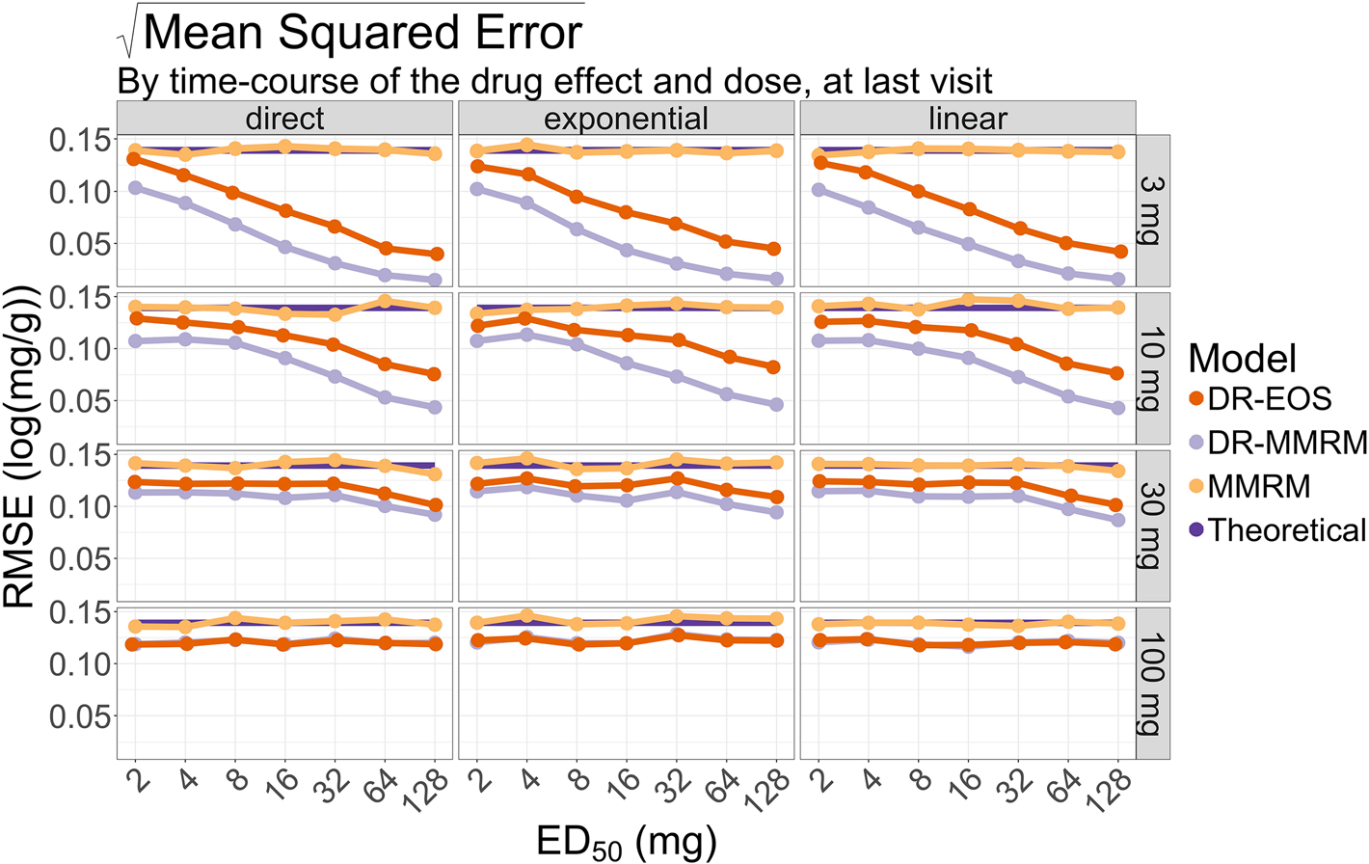
The RMSE for the investigated models with varying ED_50_, stratified by time-course of the drug effect and the dose levels. The theoretical RMSE following the study design is also shown

In Fig. 4, the RMSE for the last visit is shown, stratified by time-course of the drug effect and the dose levels. As stated in Eq. 3, this is a composite measure of variance and bias, and all methods had constant RMSE across different values of ED_50_ for the highest dose, where conventional MMRM was slightly higher than the other methods. The conventional MMRM always had a relatively constant RMSE for different time-courses of the drug effect as well as dose levels, which was higher than the dose-response informed methods and on par with the theoretical expected RMSE, $\frac{\sqrt{\omega^2+{\upsigma}^2}}{\sqrt{n}}\times \sqrt{2}\approx 0.14\ \log \left(\frac{mg}{g}\right)$. The dose-response informed methods had decreasing RMSE with increasing ED_50_ for the lower doses, dose-response MMRM RMSE was lowest at a dose of 3 mg and ED_50_ of 128 mg – the lowest dose and highest ED_50_. The DR-MMRM had the lowest RMSE in all cases.

**Fig. 5:**
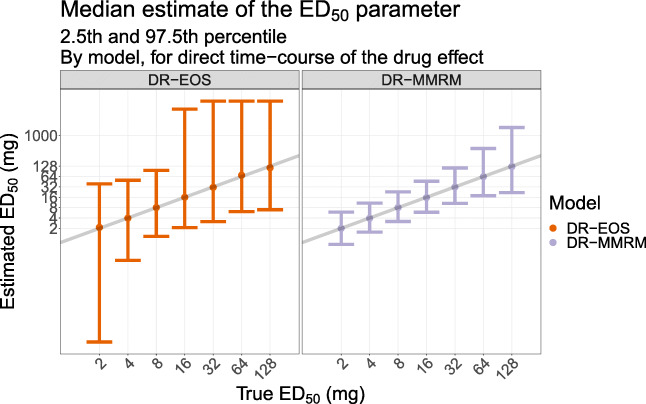
The median estimated ED_50_ with 2.5th and 97.5th percentiles vs. true ED_50_ for dose-response on end-of-study data and dose-response MMRM, exemplified by a direct time-course of the drug effect

In Fig. 5, the median estimates of ED_50_ from DR-EOS and DR-MMRM are shown, exemplified with direct time-course of the drug effect. The median estimates were generally well aligned with the true value that was used for simulation, except for the two highest ED_50_ (64 and 128 mg), where a slight bias could be seen and the uncertainty of the estimates were higher. The median was used since the mean was heavily influenced by estimates that were on the upper boundary. The estimates for all time-courses of the drug effect are shown in Appendix Fig. 3.

**Fig. 6:**
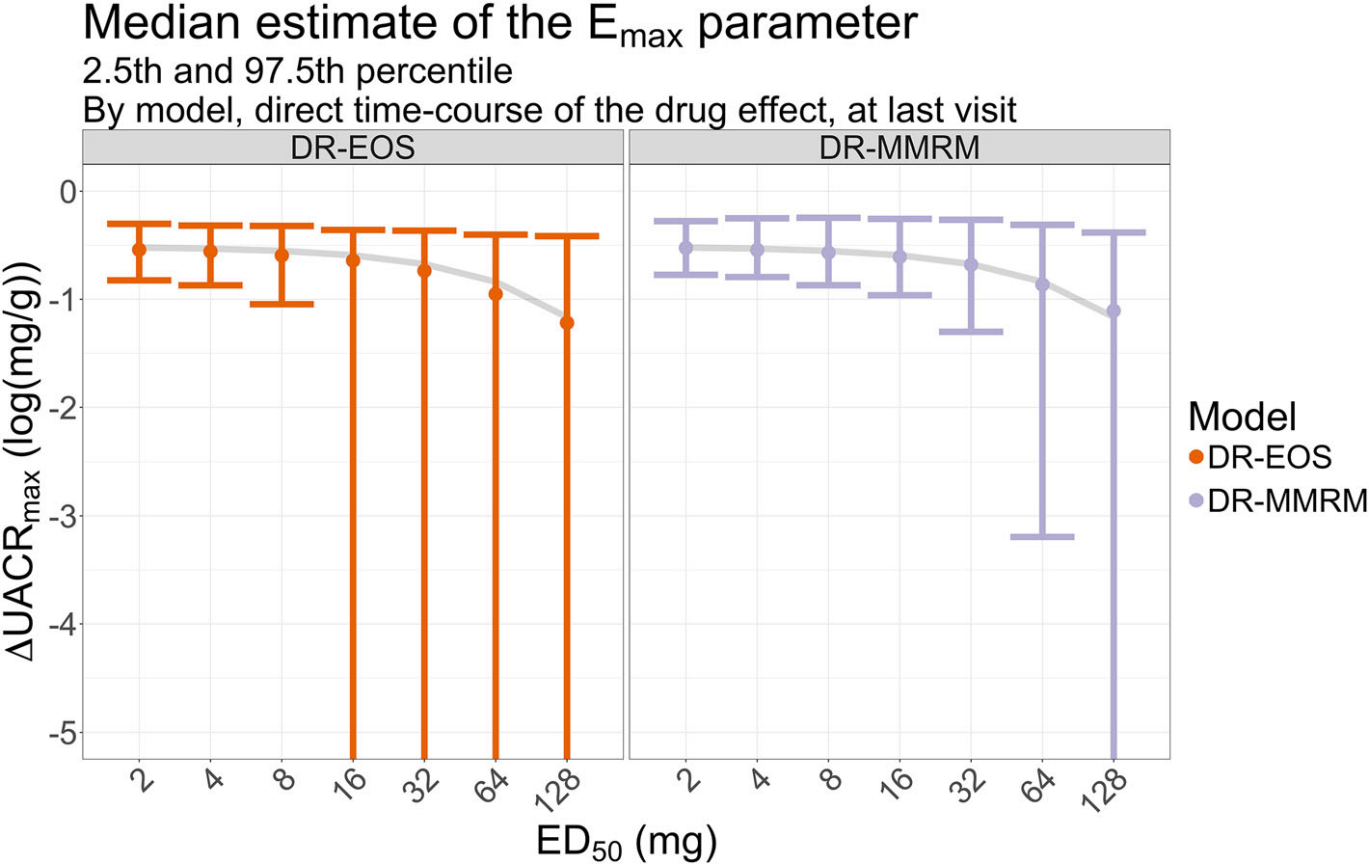
The median estimate of the E_max_ parameter with 2.5th and 97.5th percentiles for dose-response on end-of-study data and dose-response MMRM, stratified by time-course of the drug effect and ED_50_. The true E_max_ is also shown in the gray line. The y axis was cut at −5 for visibility, error bars for higher true ED_50_ extend well below the range of the graph

In Fig. 6, the median estimates of the E_max_ parameter for the last visit from DR-EOS and DR-MMRM are shown, exemplified with direct time-course of the drug effect. The 2.5th and 97.5th percentiles of the estimates are shown with error bars, which indicate that the uncertainty of the estimates was increasing with increasing ED_50_. The median was used for plotting since some extreme values influenced the mean to overestimate the E_max_ parameter for both methods. Instead, the median E_max_ was overestimated by DR-EOS but appeared unbiased for DR-MMRM. The percentiles were always wider for DR-EOS than DR-MMRM, however that may be a feature of the constraints in the settings as many values were at the upper boundary. The estimates for all time-courses of the drug effect are shown in Appendix Fig. 4.

In the simulation scenario with 3 doses, at least one of the 3 parameterizations converged successfully for all 21,000 simulated studies and provided an estimate of ED_50_ and E_max_. When 4 dose arms were used, it was possible to get an estimate of ED_50_ and E_max_ for all cases, except 2 of the 21,000 simulated studies where none of the 4 parameterizations converged. The failed attempts both occurred for an ED_50_ of 128 mg, with either exponential or direct time-course of the drug effect. For DR-EOS and conventional MMRM, estimation was always successful. A summary of the pros and cons of the investigated methods is shown in Table 1.

## Discussion

Conventional MMRM analyses are inherently unbiased if dropout is at random, which is not the case for the dose-response informed methods. None of the investigated methods had a large bias at any point – it was never more than 6% of the maximal drug effect on UACR. The DR-MMRM had lower bias than the DR-EOS, which can be explained by the fact that they use different amounts of data. The MMRM methods were also mostly within the expected range of bias following the study design and simulation setup, while the DR-EOS had a clear trend of increasing bias when ED_50_ increased.

Dose-response at end-of-study or dose-response MMRM offers an improvement in precision over conventional MMRM analyses. The improvement is mostly seen for lower doses. The precision for the highest dose is comparable for all methods, but slightly higher for MMRM. Since the precision for the highest dose level was not improved to any large degree by adding the 4th dose (3 mg), it seems that the highest dose contains most information regarding the dose-response, and certainly so with increasing ED_50_, as lower doses are not informing as much about the E_max_ and ED_50_ parameters. As expected, DR-EOS performs well (on par with DR-MMRM in terms of precision for the highest dose) since there was no dropout in the simulated data. Should we have included dropout, we know an ANCOVA with LOCF, which resembles the DR-EOS, will be biased [4, 5]. The relatively frequent observations in this study favors the DR-MMRM method as more information regarding ED_50_ is utilized.

For the correlation of residuals most conventional MMRM analyses estimate an unconstrained covariance matrix whereas we used a 1st order autoregressive covariance matrix in this work. However, again since we simulate without drop-out, this does not alter the comparisons presented here unless the variances would have varied substantially across visits.

DR-MMRM always had lower RMSE than MMRM. RMSE is the weighted measure of both precision and bias but in this example the bias was very low for all methods investigated, so RMSE was effectively a measure of the precision. Higher precision, especially in the lower dose range, allows for better predictions and interpolations to doses that have not been studied. Phase 3 clinical trials failures are predominantly due to lack of efficacy or safety concerns [21]. Choosing the correct dose could increase the success rates, which is why proper characterization of dose-response relationships are of high importance. Phase 2 trials typically aim to both establish proof-of-concept (PoC) as well as to characterize the dose-response relationship, but the number of dose arms is often limited so that the latter part is difficult. Uncertainty in the dose-response means that assumptions regarding the response in phase 3 need to be made, which is a risk that is preferably minimized. The positive trade-off between precision and bias (lowered RMSE) is encouraging and shows that the DR-MMRM methodology should be considered when evaluating dose-response in dose-finding trials when the endpoint is a repeated measure. Even when the simulated ED_50_ was low there was a marked reduction in RMSE in favor of DR-MMRM, which only grew with increasing ED_50_.

In this work the highest simulated ED_50_ was 128 mg, while the highest dose was 100 mg, i.e. well below the ED_50_. For this scenario, the dose range was not sufficient to characterize an E_max_ relationship in dose-response. This is evident from Figs. 5 and 6, where the uncertainty of the estimated ED_50_ and E_max_ parameters increases as the simulated ED_50_ increases. The limited dose range in combination with the selected model (E_max_) is the root cause for this bias when the simulated ED_50_ is high. A linear or perhaps log-linear model would likely have performed equally well in these cases. Model averaging could have been applied to avoid selection bias, which uses a weighted average of several proposed structural models [22]. That could be a future extension to this work, together with further investigations of the impact of model misspecification.

As previously discussed, it is important to choose doses wisely in the design of phase 2 studies, so that the dose-response relationship can be properly characterized, i.e. studying a sufficient number and wide enough range of doses to avoid bias. In this example there was roughly a 3-fold difference between 3 or 4 consecutive doses (4 or 5 including placebo). As clinical trials are usually optimized for sample size, this fold difference could be increased to explore a larger range with fewer doses, depending on earlier knowledge of a compound’s effectiveness. It is worth to mention that the ratio between ED_90_ and ED_10_ is 81 under an E_max_ function without sigmoidicity, a range that is seldom covered by phase 2 trials. If the confidence in a proposed ED_50_ is high, the fold difference may not need to be as wide. In this exercise we simulated without sigmoidicity in the E_max_ equation so that practically any dose larger than 0 would illicit a detectable effect, meaning that all doses carried some information on the dose-response relationship. When the true relationship is sigmoidal, further doses might be warranted to estimate ED_50_ at any meaningful certainty if there is no/little previous knowledge to learn from. Also, E_max_ and ED_50_ are correlated parameters, and it can be seen that they are biased in the same direction. Again, the setup without sigmoid E_max_ relationship makes this finding expected.

The time-course of the effect is not affecting the conclusions, because the same trends are visible in all the scenarios. The simulations were performed so that the full effect was reached at least by the end of the study (at visit 12).

One potential caveat of the MMRM methods is that they handle time as discrete values/fixed effects – each visit is independent of all other visits. If the study protocol is not followed, e.g. if a sample is taken on the wrong date, this can lead to a bias. However, in phase 2 trials such as those we have investigated in this work, subjects are typically followed closely and the study protocol adhered to. But when planned and actual visits deviate from one another there will be benefits of modeling time continuously. For the conventional MMRM method, the information about a dose level in relation to other doses is also not considered, which DR-MMRM amends.

As the same model (E_max_ without sigmoidicity) was used for both simulation and estimation, the results are possibly different in terms of power, precision and accuracy than they would be if another model had been used for simulation (e.g. simulating with sigmoidicity but estimating without, or an even more misspecified model). It should be noted that if a sigmoid model had been used for both simulation and estimation, the comparison with conventional MMRM probably would be less favorable. However, this does not affect the conclusions regarding DR-MMRM versus DR-EOS. Also, both the DR-EOS and DR-MMRM were implemented with the same dose-response model to make a fair comparison. The same argument as before can be reiterated regarding the number of doses – more doses could have been studied but would likely not alter this comparison. The dose-response was equally well characterized already with three doses and, besides, it is uncommon to have a large number of doses in phase 2 trials.

The drug effect was simulated to give a rather large (40%) reduction in UACR, which is more than the 30% reduction recently suggested as a meaningful clinical improvement [16, 23]. This affects the sample size in this setup so that studies are smaller than they would be in reality, but again, would not alter the comparison of the investigated methods.

## Conclusions

All three methods (dose-response on end-of-study data, conventional MMRM and dose-response MMRM) predicted the outcome at the end of the study with good accuracy. When MMRM methods are warranted, for instance when there is a highly variable placebo response, the precision of MMRM models could be improved by adding a dose-response relationship. The precision was mostly improved for the lower dose arms. This method can aid in the general understanding of the dose-response characteristics of a compound and increase confidence in the dose(s) to bring forward to the next stage in drug development.

## Electronic supplementary material

ESM 1 (PDF 29 kb)

ESM 2 (DOCX 802 kb)

ESM 3 (DOCX 579 kb)

ESM 4 (DOCX 572 kb)

ESM 5 (R 14 kb)
