# Supplementary figures and images for: Dose-Response Mixed Models for Repeated Measures – a New Method for Assessment of Dose-Response
Source: Pharm Res. 2020 Jul 31;37(8):157. doi: 10.1007/s11095-020-02882-0 (PMC7651607; doi:10.1007/s11095-020-02882-0)

## Simulation and estimation setup

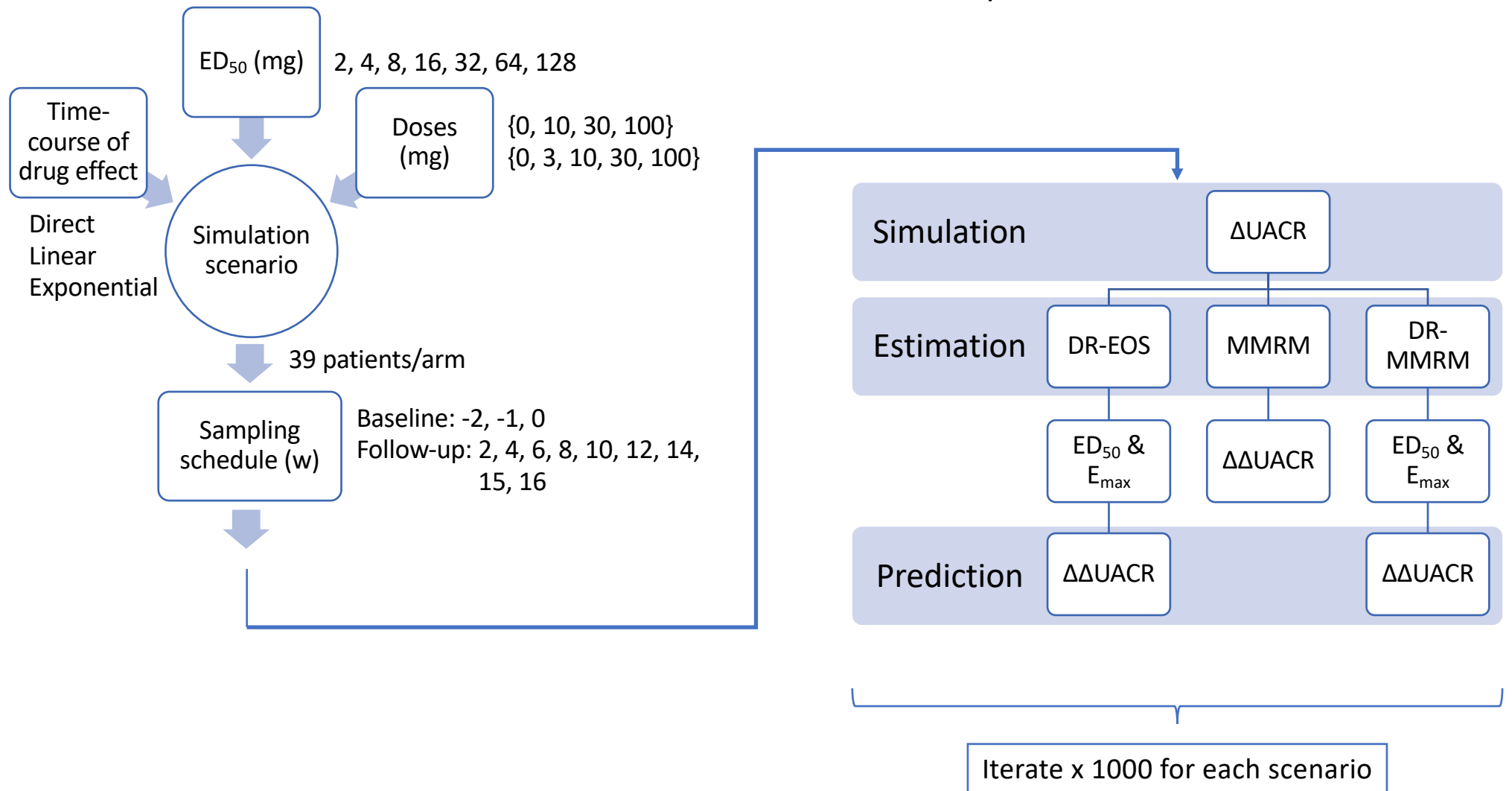

Supplement: Supplementary file 1 — (PDF 29 kb) [file 11095_2020_2882_MOESM1_ESM.pdf]
