# Supplementary material for: Dose-Response Mixed Models for Repeated Measures – a New Method for Assessment of Dose-Response
Source: Pharm Res. 2020 Jul 31;37(8):157. doi: 10.1007/s11095-020-02882-0 (PMC7651607; doi:10.1007/s11095-020-02882-0)
Supplement: Supplementary file 2 — (DOCX 802 kb) [file 11095_2020_2882_MOESM2_ESM.docx]

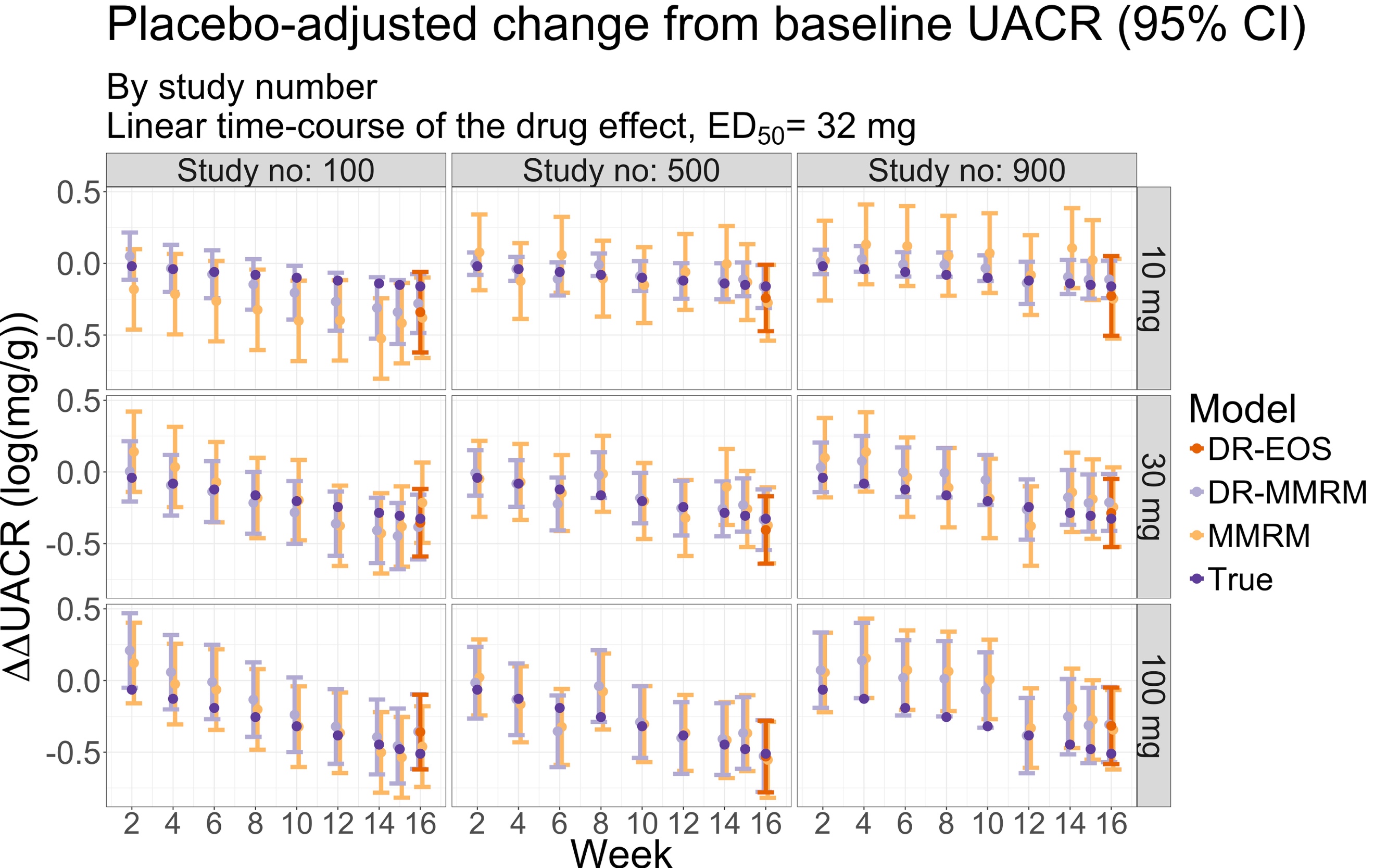
Appendix Figure 1. Placebo-adjusted ∆UACR and 95% CI for 3 studies with linear time-course of the drug effect where ED_50_ = 32 mg, stratified by dose (simulations with 3 doses and placebo).
