# Supplementary material for: Dose-Response Mixed Models for Repeated Measures – a New Method for Assessment of Dose-Response
Source: Pharm Res. 2020 Jul 31;37(8):157. doi: 10.1007/s11095-020-02882-0 (PMC7651607; doi:10.1007/s11095-020-02882-0)
Supplement: Supplementary file 3 — (DOCX 579 kb) [file 11095_2020_2882_MOESM3_ESM.docx]

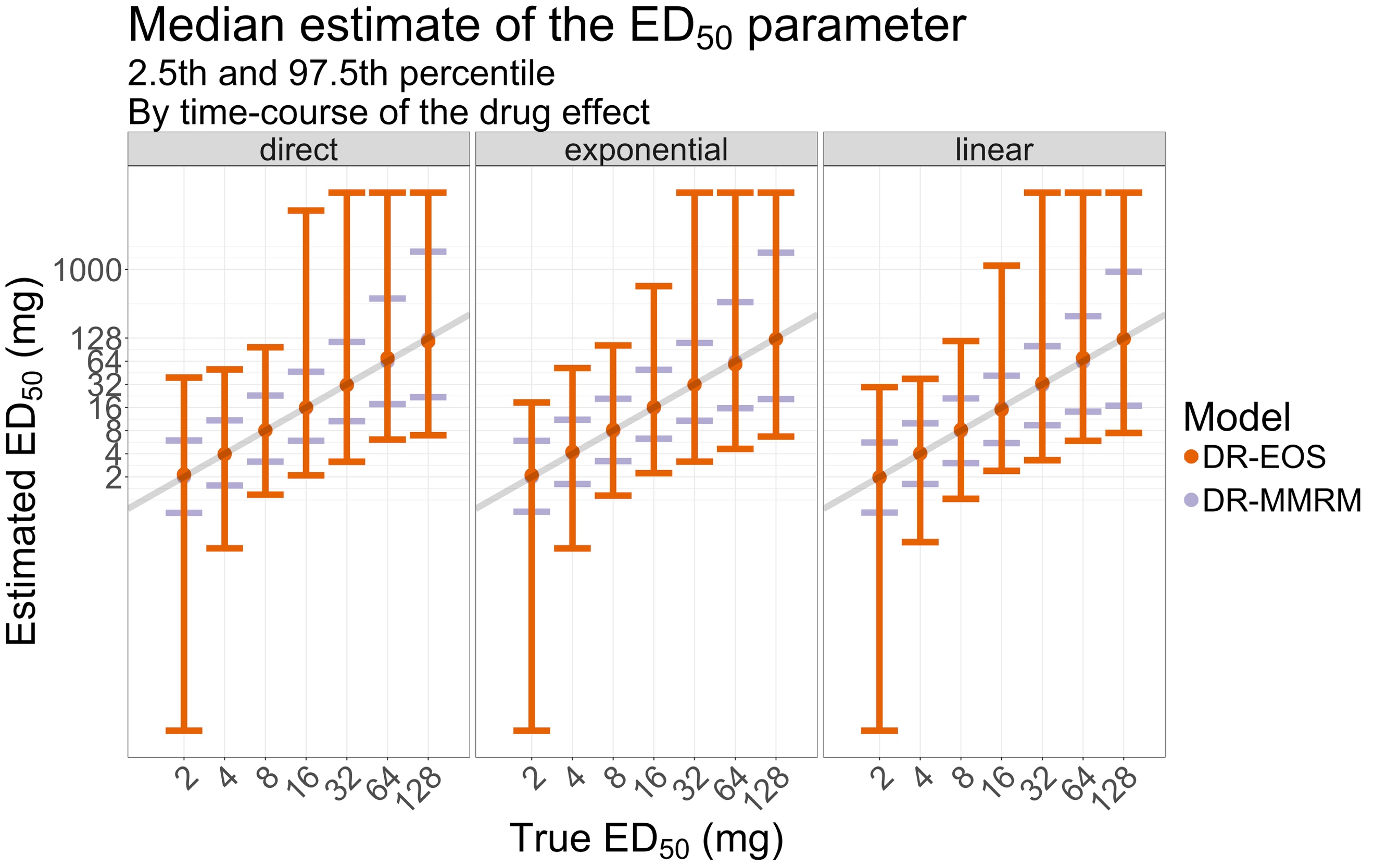
Appendix Figure 2. The median estimated ED_50_ with 2.5^th^ and 97.5^th^ percentiles vs. true ED_50_ for dose-response on end-of-study data and MMRM with dose-response, stratified by the time-course of the drug effect.
