# Supplementary material for: Dose-Response Mixed Models for Repeated Measures – a New Method for Assessment of Dose-Response
Source: Pharm Res. 2020 Jul 31;37(8):157. doi: 10.1007/s11095-020-02882-0 (PMC7651607; doi:10.1007/s11095-020-02882-0)
Supplement: Supplementary file 4 — (DOCX 572 kb) [file 11095_2020_2882_MOESM4_ESM.docx]

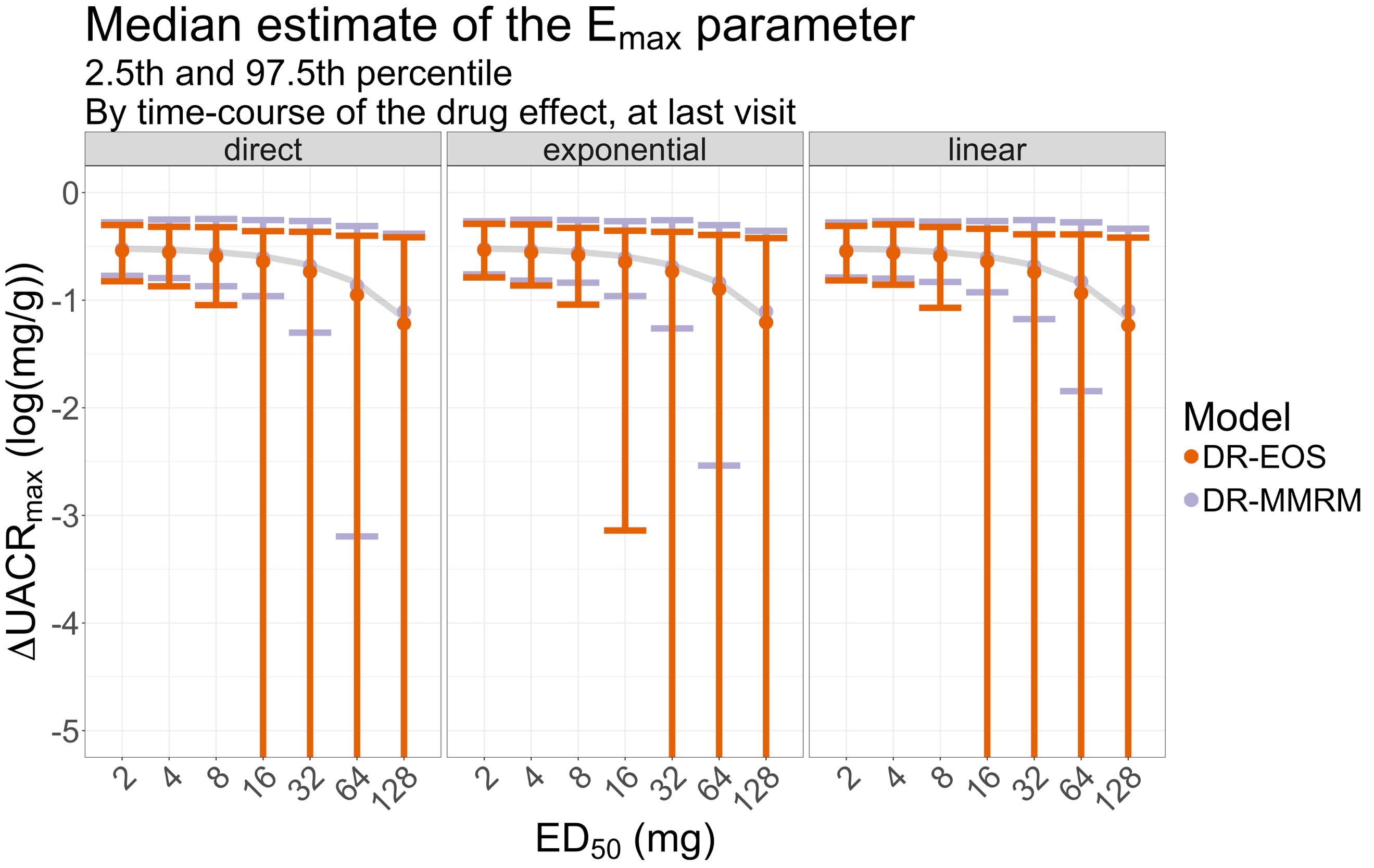


Appendix Figure 3. The median estimate of the E_max_ parameter and 2.5^th^ and 97.5^th^ percentiles for dose-response on end-of-study data and MMRM with dose-response, stratified by time-course of the drug effect and ED_50_. The true E_max_ is also shown in the gray line. The y axis was cut at -5 for visibility, error bars for higher true ED_50_ extend well below the range of the graph.
